# Pulling the genome in opposite directions to dissect gene networks

**DOI:** 10.1186/s13059-018-1425-1

**Published:** 2018-03-26

**Authors:** Charles A. Gersbach, Rodolphe Barrangou

**Affiliations:** 1 Department of Biomedical Engineering, Center for Genomic and Computational Biology, Duke University, Durham, NC USA; 2 Department of Orthopaedic Surgery, Duke University Medical Center, Durham, NC USA; 3 Department of Food, Bioprocessing and Nutrition Sciences, North Carolina State University, Raleigh, NC 27695 USA

## Abstract

Orthogonal CRISPR-Cas systems have been integrated into combinatorial screens to decipher complex genetic relationships in two recent studies.

## Introduction

Genetic screens have been the lifeblood of forward genetics. They have enabled widespread discoveries of gene function, leading to meaningful advances in medicine, biotechnology, and agriculture. Nevertheless, technologies for performing these screens have been limited by scale, specificity, and targeting range of tools for investigating and perturbing the genome [1]. Recently, clustered regularly interspaced short palindromic repeats (CRISPR)-based screens with libraries of guide RNAs (gRNAs) have revolutionized the power of genetic screens by overcoming many of these limitations with knockout, repression, and activation screens of both the coding and non-coding genome [2]. Two recent studies from Boettcher et al. [3] and Najm et al. [4] exponentially increase the power of these screens by integrating orthogonal CRISPR-Cas systems into combinatorial screens, demonstrating the potential to expand and combine these methods to decipher complex genetic relationships. By exploiting orthogonal Cas9 proteins from the CRISPR toolbox, these studies show how a combinational approach provides flexibility and potential to scale for more sophisticated and elaborate next-generation screens.

## CRISPR-based genetic screens

Building on the experience of more than a decade of lentiviral shRNA-based screens [1], the original CRISPR-based screens capitalized on high-throughput synthesis of DNA oligonucleotides encoding gRNA targeting sequences that could be readily packaged into a lentiviral vector [2]. The resulting pools of lentiviral vectors can be quantifiably dosed and delivered to a population of cells such that each cell receives a single gRNA. Consequently, if that pool of cells also expresses the RNA-guided endonuclease Cas9, each cell receives a unique genetic perturbation specifically determined by the gRNA targeting sequence. That population of cells can then be selected for the gain or loss of specific phenotypic properties, and the unique gRNA sequences within those selected cells can be identified by next-generation sequencing. By mapping those gRNAs back to their genomic target sites, it is possible to determine genes or genomic regions that are involved in and responsible for modulating the selected cellular phenotype.

The early CRISPR screens focused on gene knockouts, using gRNAs targeted to the coding regions of genes in combination with the commonly used *Streptococcus pyogenes* Cas9 endonuclease (SpyCas9) [5, 6]. However, as the CRISPR toolbox grew, diversified, and matured, so did the varieties of CRISPR screens. Libraries of gRNAs targeted to gene promoters, in combination with repression by CRISPR interference (CRISPRi) and CRISPR activation (CRISPRa) variants of the nuclease-deactivated Cas9 (dCas9), enabled screens based on phenotypes that result from decreased or increased gene expression, rather than gene knockout [7, 8]. This later expanded to screens of the non-coding genome, using either gene editing with Cas9 to knockout or delete gene regulatory elements, or epigenome editing with dCas9-based tools for loss- or gain-of-function of regulatory activity [9]. Several recent studies have overcome a number of technical challenges to deliver defined pairs of gRNAs together, thus enabling the screening of phenotypes based on combinations of perturbations that reveal relationships between genes and/or non-coding sequences. However, these screens used a single Cas9 enzyme, and thus both perturbations were uni-dimensional and co-directional (i.e., gene knockout) and all gRNAs recruited the same Cas9 effector (i.e., SpyCas9) to their target site. Given the importance of interactions between genetic elements in controlling and regulating complex cellular networks and functions, it is necessary to assess these relationships rather than investigate sequences one at a time, including sometimes re-orienting effects in opposite directions.

## Combinatorial, bi-directional screens with multiple CRISPR effectors

**Fig. 1:**
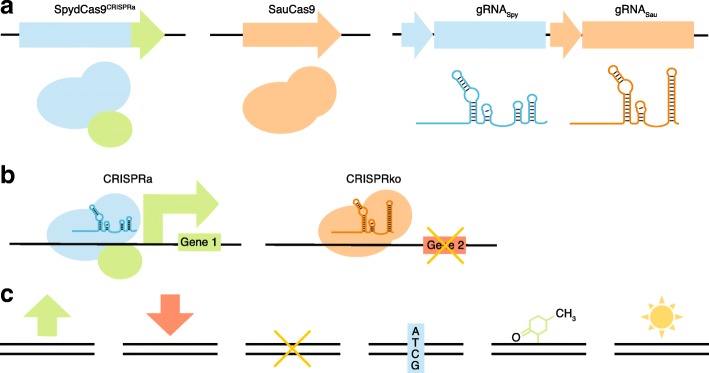
Boettcher et al. [3] and Najm et al. [4] demonstrate combinatorial bi-directional CRISPR screens integrating gene activation and gene knockout platforms. **a** The dual guide RNA (*gRNA*) expression cassettes are synthesized on arrays with pools of gRNAs compatible with SpydCas9 and SauCas9 that target a distinct set of gene promoters and gene coding sequences, respectively. **b** Each cell is engineered to express both a SpydCas9 activator and the SauCas9 nuclease, and also receives a single dual gRNA cassette, leading to activation and knockout of a unique gene pair. The pool of cells with diverse gRNA pairs is selected based on unique phenotypes conferred by these divergent gene perturbations, which are identified by sequencing the gRNA cassettes. **c** Various combinations of orthogonal Cas9 effectors enable concurrent control of transcriptional activation, repression, knockouts, base editing, epigenome alteration, and/or imaging

Boettcher et al. [3] and Najm et al. [4] have reported the first examples of pushing past this challenge by integrating two orthogonal CRISPR-Cas9 systems into pooled screens (Fig. 1). Arguably, one of the most exciting and enabling prospects of the CRISPR-Cas9 technology is the ability to induce gain-of-function perturbations with CRISPRa or deposition of other activating epigenetic marks, in contrast to earlier technologies like RNA interference that were only capable of loss-of-function perturbations. Boettcher et al. [3] take advantage of this potential by combining orthogonal CRISPRa screens with the more conventional CRISPR knockout screens [3]. A central challenge to combining CRISPR-based loss-of-function and gain-of-function screens is targeting the CRISPRa tools with one set of gRNAs and the Cas9 nuclease for gene knockout with a distinct set of gRNAs, and avoiding any cross-reactivity between these components. Boettcher et al. [3] accomplish this by using SpyCas9 with the CRISPRa SunTag system and the Cas9 nuclease from *Staphylococcus aureus* (SauCas9) for gene knockout. SpyCas9 and SauCas9 recognize distinct protospacer-adjacent motif (PAM) targeting sequences. Moreover, they have no detectable gRNA cross-reactivity as their gRNAs are solely and specifically recognized and loaded into their respective Cas9 protein due to their distinct gRNA sequence and structure. A lentiviral vector was designed to carry a single pair of SpyCas9 and SauCas9 gRNAs to each cell.

The orthogonal combinatorial screen was tested in the context of evaluating modifiers of sensitivity to treatment with the tyrosine kinase inhibitor imatinib in the human chronic myeloid leukemia cell line K562. As a demonstration of the power of gain-of-function screens, the genome-wide CRISPRa screen alone identified 332 genes of which increased expression modulated sensitivity to imatinib. Of these genes, 21% are not normally expressed in K562 cells and thus would not have been recovered by a loss-of-function perturbation, illustrating the advantages of this approach. For the combinatorial screen, Boettcher et al. [3] targeted 87 of the hits from this primary CRISPRa screen with 174 SpyCas9 gRNAs, along with 11,594 SauCas9 gRNAs targeting 1327 genes involved in cancer-relevant signaling pathways, for a total of 115,449 distinct genetic interactions. Therefore, this screen was designed to identify cancer-relevant genes that when knocked out enhance or diminish the effect of activation of the 87 genes from the primary screen. This led to the identification of several dependencies, including one in which the cancer cells became susceptible to treatment with a drug targeting the product of one gene only when a second gene had also been knocked out. This further illustrates the need to use a combinatorial approach to unravel interactions between genetic elements involved in complex phenotypes.

Likewise, Najm et al. [4] optimized an approach to combine orthogonal screens with SpyCas9 and SauCas9 [4]. They first determined an algorithm for optimal SauCas9 gRNA design for highly efficient gene knockout, building on previous similar work they had published for SpyCas9 [10]. Using these optimal gRNAs, they performed synthetic lethal screens with both SpyCas9 and SauCas9 nuclease for paired knockouts of genes involved in apoptosis. Extensive characterization of screening results suggested significantly enhanced robustness and reproducibility compared with earlier combinatorial approaches. To explore the potential of orthogonal screens with distinct perturbations, they used the SpyCas9-VPR CRISPRa system to activate expression of 38 different oncogenes, along with SauCas9 targeted to knockout 45 tumor suppressors. Three gRNAs were used for each gene, for a total of 1710 genetic interactions with 15,390 gRNA pairs. The effect of the gRNA pairs on cell proliferation was assessed following 21 days of growth of HA1E cells, in which p53 tumor suppressor activity is suppressed by immortalization with the large T antigen. Several known and novel genetic interactions were identified in which the lethal effects of activation of a tumor suppressor were muted by activation of an oncogene, or conversely the proliferative effects of oncogene activation were lessened by tumor suppressor knockout.

## Outlook and future directions

The potential for dissecting genetic interactions with complementary gain- and loss-of-function screens are diverse and exciting. Both Boettcher et al. [3] and Najm et al. [4] focused on cancer cell growth fitness as a first proof-of-principle, but future studies may incorporate more advanced analysis of complex drug combinations to find novel therapeutic regimens. Additionally, there is a rich potential to use this approach to investigate gene networks that drive other complex cell phenotypes and functions, including pluripotency, differentiation, reprogramming, migration, and cell–cell interactions. Moreover, using this approach to decipher complex regulatory logic of the non-coding genome is a particularly compelling future application of these technologies [9].

While orthogonal gene activation and knockout screens fill an important and obvious technological gap, the diversity of genome engineering functions made possible by CRISPR genome and epigenome editing tools opens the door to many other perturbations, and combinations thereof. Any combination of targeted knockout, base editing, activation, repression, DNA methylation/demethylation, histone modifications, or even forced chromatin looping is readily possible. Scenarios can be envisioned in which multiplexing more than two orthogonal screens could be achieved, which will be facilitated by increased mining and characterization of diverse and orthogonal CRISPR-Cas systems. Indeed, there is much natural diversity within and between CRISPR types and subtypes that can be exploited.

Biology has evolved enormous complexity through combinatorial diversity of many types of molecular interactions. The only hope to decipher this complexity is to develop precise molecular tools that match this diversity, and enable the dissection and perturbation of complex biological systems. The expansion of the CRISPR toolbox, in combination with advances in library synthesis and viral vector delivery tools, ensures the continuation of the CRISPR revolution and catalyzes our progress along this quest.

## Abbreviations

Cas: CRISPR-associated
CRISPR: Clustered regularly interspaced short palindromic repeats
CRISPRa: CRISPR activation
dCas9: Nuclease-deactivated Cas9
gRNA: Guide RNA
